# Ketamine modulates fetal hemodynamic and endocrine responses to umbilical cord occlusion

**DOI:** 10.14814/phy2.12962

**Published:** 2016-09-05

**Authors:** Miguel A. Zarate, Eileen I. Chang, Andrew Antolic, Charles E. Wood

**Affiliations:** ^1^ Department of Physiology and Functional Genomics University of Florida College of Medicine Gainesville Florida; ^2^ Department of Pharmacodynamics University of Florida College of Pharmacy Gainesville Florida

**Keywords:** Endocrine, fetal hypoxia, hemodynamics, umbilical cord occlusion

## Abstract

Umbilical cord occlusion (UCO) is a hypoxic insult that has been used to model birth asphyxia and umbilical cord compression in utero. UCO triggers vigorous neural and endocrine responses that include increased plasma ACTH and cortisol concentrations, increased blood pressure (BP), and decreased heart rate (HR). We have previously reported that ketamine, a noncompetitive N‐methyl‐D‐aspartate receptor antagonist, can modify the fetal hemodynamic and ACTH responses to ventilatory hypoxia and cerebral ischemia‐reperfusion. We performed the present experiments to test the hypothesis that ketamine has similar effects on the neuroendocrine and cardiovascular responses to UCO. Fetal sheep were chronically catheterized at gestational day 125. Ketamine (3 mg/kg) was administered intravenously to the fetus 10 min prior to the insult. UCO was induced for 30 min by reducing the umbilical vein blood flow until fetal P_a_O_2_ levels were reduced from 17 ± 1 to 11 ± 1 mm Hg. UCO produced an initial increase on fetal BP in both control and ketamine groups (*P *=* *0.018 time), followed by a decrease in the control group, but values remained higher with ketamine. HR decreased after UCO (*P *=* *0.041 stimulus*time) in both groups, but the reduction was greater initially in control compared to ketamine groups. Fetal P_a_ CO_2_ levels increased after UCO (*P *<* *0.01 stimulus*time), but values were higher in the control versus ketamine groups. UCO significantly decreased fetal pH values (*P *<* *0.01 stimulus*time) with a greater effect on the control versus ketamine group. Ketamine delayed the cortisol responses to UCO (*P *<* *0.001 stimulus*time), and UCO produced a robust increase in ACTH levels from 19 ± 2 to 280 ± 27 pg/mL (*P *<* *0.001 stimulus*time), but there were no differences in ACTH levels between UCO groups. We conclude that ketamine augmented the cardiovascular response to UCO, but did not alter the ACTH response to UCO.

## Introduction

Transient umbilical cord occlusion (UCO) produces global systemic asphyxia and reoxygenation, which might influence fetal development, produce CNS damage, and lead to morbidities later in life (Calkins and Devaskar 2011; Castillo‐Melendez et al. 2013). UCO has been used to model hard labor or abnormal positioning of the umbilical cord, and has been a useful tool for the study of brain damage caused by birth asphyxia (Castillo‐Melendez et al. 2004, 2013; Volpe 2009). Hypoxemia stimulates the carotid chemoreceptors, resulting in robust sympathetic autonomic and neuroendocrine responses that favor fetal survival of the insult. Cardiovascular responses include reduction in heart rate and redistribution of combined ventricular output (CVO) toward relevant organs such as the brain, heart, adrenal glands, and umbilical vascular beds (Sidi et al. 1983; Giussani et al. 1993).

An important component of the fetal response to stress is the increased activity of the hypothalamus–pituitary–adrenal (HPA) axis (Wood and Walker 2015). Cortisol is important for maturation of the late‐gestation fetus and for survival of stress (Wood and Walker 2015). Hypoxic hypoxia (Boddy et al. 1974; Giussani et al. 1994; Chang and Wood 2015), asphyxic hypoxia (Roelfsema et al. 2005), ischemic hypoxia (Powers and Wood 2007), acidemia (Wood and Chen 1989), and hypercapnia (Chen and Wood 1993) stimulate fetal ACTH and cortisol secretion. We have reported that ketamine, a clinically useful drug that blocks N‐methyl‐D‐aspartate (NMDA) receptors (Mishra et al. 2001), partially inhibits the fetal ACTH response to hypoxic hypoxia (Chang and Wood 2015), and almost completely inhibits the fetal ACTH response to brachiocephalic artery occlusion (a model of brain and carotid body ischemic hypoxia) (Powers and Wood 2007). Furthermore, we have demonstrated that intravenous injection of NMDA stimulates fetal ACTH secretion, and that the effect is partially dependent on prostaglandin biosynthesis (Knutson and Wood 2010). Boekkooi et al. (1995) reported that ketamine interrupts the chemoreflex control of the heart rate in the late‐gestation fetal sheep. Based on the assumption that the fetal HPA axis responses to the various forms of hypoxia are mediated by the fetal carotid chemoreceptors, we hypothesized that ketamine would reduce the magnitude of the fetal HPA response to UCO. This study was designed to test this hypothesis.

## Materials and Methods

All the experiments were approved by the University of Florida Animal Care and Use Committee and conducted in accordance with the Guiding Principles for Use of Animals of the American Physiological Society. The study involved 34 (*n* = 5–11/group) chronically catheterized fetal sheep (125 ± 4 gestation days). Late‐gestation pregnant ewes had mixed breeds, and had ad libitum access to food and water.

### Fetal surgery

Surgical procedures were performed between 115 and 130 days of gestation. Fetal sheep were chronically catheterized as previously described by Wood and Saoud (1997). Briefly, ewes were fasted for 24 h, and received preoperative care consisting of blood analysis, and the administration of ampicillin (Polyflex ^®^, Boehringer Ingelheim VetMedica Inc., St. Joseph, MO) preceding anesthesia induction with 0.5–2% isoflurane with oxygen. We surgically placed vascular catheters in both femoral arteries and veins, and a catheter in the amniotic fluid space. A 12‐mm extravascular occluder (In Vivo Metric, Healdsburg, CA) was placed around the umbilical cord. Following fetal catheterization, catheters were placed in maternal femoral arteries and veins. All catheters and the extravascular occluder exited the ewe from the right flank and were placed into a disposable pouch. After the surgical procedures, the ewe was allowed to recover for a minimum of 5 days and received postoperative care. This period of time was necessary to considerably reduce any maternal effect.

### In vivo experimental procedures

During the experiment, the ewes were conscious and freestanding in their pens. For each experiment, fetal mean arterial blood pressure (BP) and heart rate (HR) were recorded continuously using standard pressure transducers (Abbott Labs, Abbott Park, IL), a laptop computer with custom‐written National Instruments LabView software, and a National Instruments Compact DAQ signal conditioner and analog‐to‐digital conversion hardware. Fetal femoral arterial BP was measured and corrected by subtracting the amniotic fluid pressure. BP and HR were recorded prior to and following the administration of ketamine intravenously (3 mg/kg, 10 min prior to UCO or sham UCO). UCO was accomplished by infusion of saline into the extravascular occluder. The degree of occlusion was adjusted so as to lower P_a_O_2_ approximately 50% for 30 min. Sham UCO consisted of manipulation of the catheters and occluder, without infusion of saline into the occluder. Fetal and maternal blood samples (5 mL) were collected in K_2_ EDTA vacutainer tubes and heparinized syringes (1 mL) at six specific time points (−10, 0, 5, 10, 20, and 30 min after the start of UCO or sham UCO). Blood gases were monitored using a Vet Scan^®^ i‐STAT^®^ (Abaxis, Union City, CA). Blood samples were kept on ice until centrifugation of plasma (3000 g for 20 min at 4°C). After centrifugation, plasma samples were transferred into polypropylene tubes and stored at −20°C until hormone levels (ACTH and cortisol) were measured.

### Endocrine analysis

Assays were performed as previously described by Chang and Wood (2015). Samples collected during the in utero experiments were analyzed for plasma hormone measurements: ACTH concentrations were measured using radioimmunometric assay (DiaSorin, Stillwater, MN). Cortisol was measured using enzyme‐linked immunoassay (Oxford Biomedical, Oxford, MI), according to manufacturer's instructions, respectively. The sample extraction and preparation were previously described by different laboratories.

### Experimental design and statistical analysis

The experiment involved four groups of animals (±ketamine, ±UCO) in a full factorial randomized complete design. Data were analyzed by three‐way ANOVA, with stimulus (±UCO), treatment (±ketamine), and time (repeated measurements) as factors. We used the Mixed Procedure of SAS/STAT 9.3^®^ (SAS Institute Inc., Cary, NC), and determined statistical differences with the Duncan post hoc test. Data are presented as mean ± standard error of the mean (SEM), and significance was declared at *P *<* *0.05.

## Results

### Blood gases

**Figure 1:**
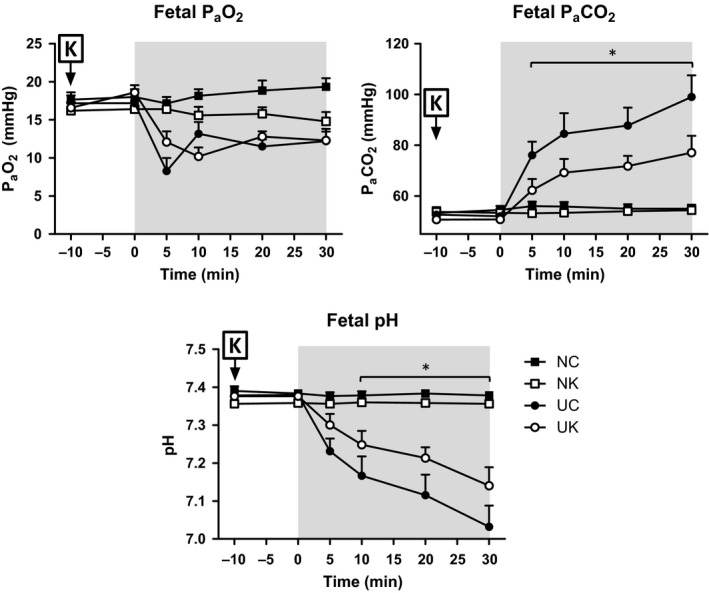
Fetal P_a_O_2_, P_a_ CO_2_, and pH prior (−10 to 0 min) and during UCO stimulation (5, 10, 20, and 30 min). (NC = normoxia control, *n* = 7; NK = normoxia ketamine, *n* = 5; UC = umbilical cord occlusion control, *n* = 11; UK = umbilical cord occlusion ketamine, *n* = 10). Asterisks (*) indicate statistical difference (*P *<* *0.05) between UC versus UK using the Duncan post hoc test. Baseline values (average of −10 through 0 min) were statistically different (*P *<* *0.01) compared to the values during the asphyxic stimulation (5, 10, 20, and 30 min) in all the variables. Data are presented as means ± SEM.

As shown in Figure 1, UCO decreased the fetal P_a_O_2_ from 17 ± 1 to 11 ± 1 mm Hg, increased P_a_CO_2_ from 53 ± 1 to 102 ± 6 mm Hg, and decreased pH_a_ from 7.38 ± 0.01 to 7.01 ± 0.04 (*P *<* *0.001, stimulus*time). Neither sham UCO nor ketamine alone had any significant effect on the fetal blood gases. After ketamine injection, UCO produced less dramatic changes in fetal P_a_CO_2_ (from 53 ± 1 to 77 ± 6 mm Hg) and pH_a_ (from 7.37 ± 0.01 to 7.14 ± 0.05). Because we were adjusting the inflation to control P_a_O_2_, the changes in this variable were not affected by ketamine. In contrast, the larger changes in P_a_CO_2_ and pH_a_ during UCO were significantly greater (*P *<* *0.001, stimulus*time).

### Fetal hemodynamics

**Figure 2:**
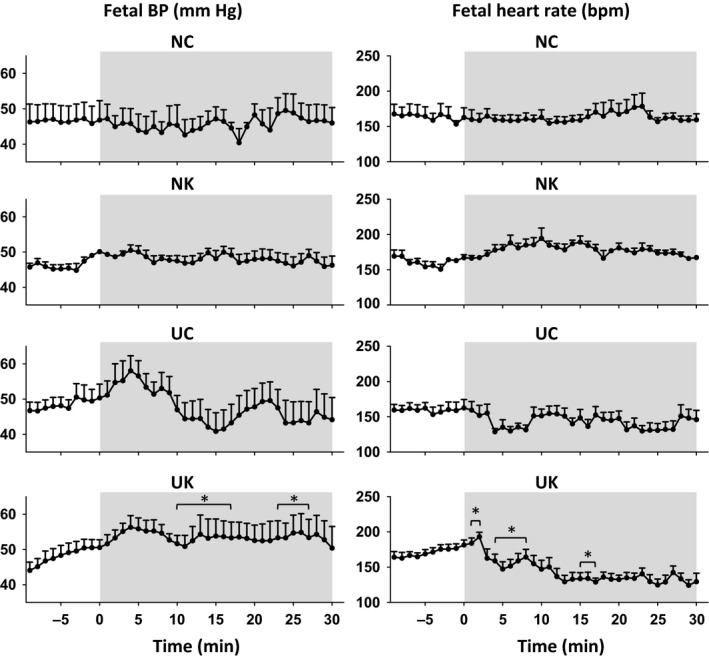
Fetal femoral BP and HR measured prior (−10 to 0 min) and during (1–30 min) UCO stimulation. Asterisks (*) indicate statistical difference (*P *<* *0.05) between UC versus UK using the Duncan post hoc test. Abbreviations are similar as in Figure 1. Fetal BP and HR values are reported as means ± SEM.

As shown in Figure 2, ketamine stimulated an increase in fetal blood pressure (from 46.0 ± 2.7 to 48.5 ± 3.0 mm Hg in NK group) and no consistent changes in fetal heart rate was observed, as analyzed in the first 10 min (pre‐UCO) of the experiment. UCO increased fetal BP (from 50.4 ± 3.8 to 58.0 ± 4.1 mm Hg in UC group and from 50.5 ± 4.1 to 56.3 ± 4.4 mm Hg in the UK group; *P *<* *0.05 stimulus*time), compared with no statistically significant change in the control group (41 ± 3 mm Hg). Fetal blood pressure remained higher during UCO after ketamine administration compared to fetuses not treated with ketamine (54.3 ± 6.3 in UK vs. 46.4 ± 5.8 mm Hg, *P *=* *0.01 stimulus*time).

UCO significantly decreased fetal HR from 162 ± 10 to 130 ± 9 beats/min. Ketamine stimulated a transient increase in fetal heart rate at the beginning of UCO (from 164 ± 10 to 181 ± 10 beats/min), followed by a prolonged enhancement of the fetal bradycardia (129 ±10 beats/min in UK group vs. 148 ± 9 beats/min in UC group) in the latter half of the UCO.

### Endocrine variables

**Figure 3:**
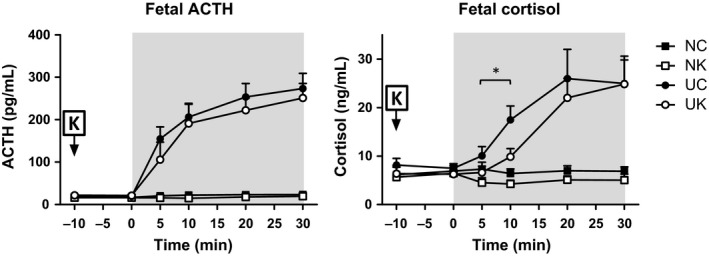
Fetal plasma concentration values of ACTH and cortisol measured prior (−10 to 0 min) and during (5, 10, 20, and 30 min) UCO stimulation. Asterisks (*) indicate statistical difference (*P *<* *0.05) between UC versus UK using the Duncan post hoc test. Baseline values (average of −10 through 0 min) were statistically different (*P *<* *0.01) compared to the values during the asphyxic stimulation (5, 10, 20, and 30 min) in all the variables. Abbreviations and number of experiments per group are reported in the legend to Figure 1. Fetal ACTH and cortisol plasma values are presented as means ± SEM.

Figure 3 showed that UCO produced a strong increase in fetal ACTH from 19 ± 2 to 280 ± 27 pg/mL (*P *<* *0.001, stimulus*time). However, ketamine did not significantly change the magnitude of the fetal ACTH response to UCO. Fetal plasma cortisol was significantly increased from 7 ± 1 to 25 ± 4 ng/mL in response to UCO. Ketamine delayed the cortisol response compared to the control group (*P *<* *0.001 stimulus*time). Nevertheless, the peak cortisol concentration was similar in the two UCO groups (24.8 ± 4.0 and 25.0 ± 4.0 in ketamine‐treated and ‐untreated groups, respectively). There were no statistically significant changes in ACTH or cortisol in two non‐UCO groups (±ketamine).

## Discussion

The results of this study suggest that ketamine does not block the HPA response to asphyxic hypoxia (UCO) in the late‐gestation fetal sheep. This is in contrast to our previous studies in which ketamine attenuated fetal ACTH responses to ischemic hypoxia (brachiocephalic occlusion) and hypoxic hypoxia (HH, maternal ventilatory hypoxia) (Powers and Wood 2007; Chang and Wood 2015). We believe that, while we had proposed that the response to all three forms of hypoxia (asphyxic, ischemic, and hypoxic) would be dominated by the carotid arterial chemoreflex and therefore reduced by ketamine, the differences in responses among these three stimuli give us insight into how ketamine interacts with the fetal response to the various forms of oxygen deprivation in utero.

Because we aimed to compare similar degrees of hypoxia between UCO and HH, we adjusted the degree of umbilical cord occlusion to control fetal P_a_O_2_. In both HH and UCO experiments, we decreased fetal P_a_O_2_ to approximately 10 mm Hg. In doing so, we aimed to match the decrease in oxygen delivery rates to the brain and allow a meaningful comparison of molecular responses within different brain regions. These two methods of hypoxia produce contrasting changes in P_a_CO_2_ and pH_a_. In HH, the hyperventilation of the ewe reduces P_a_CO_2_ and increases pH_a_ in both fetus and mother (Chang and Wood 2015). In UCO, P_a_CO_2_ is dramatically increased and pH_a_ is dramatically decreased because of reduced perfusion and gas exchange across the placenta. Results of previous experiments in this laboratory have demonstrated that changes in pH_a_ and P_a_CO_2_, caused by acidification of fetal blood or by mixing carbon dioxide into the maternal inspired gas, results in activation of the fetal HPA axis (Wood and Chen 1989; Chen and Wood 1993). While it is likely that acidemia or hypercapnia confounded the effect of ketamine on the HPA response, the results do clearly demonstrate that the multiple chemoreceptor inputs to the HPA axis are not mediated by a single common pathway.

Cardiovascular responses to UCO – increased mean arterial pressure and decreased heart rate – were consistent with results from other groups (Itskovitz and Rudolph 1987; Wassink et al. 2007). Ketamine significantly modified the fetal hemodynamic response, resulting in the higher mean arterial pressure and heart rate in UCO fetuses. Although the magnitudes of the cardiovascular effects of ketamine were small, the mechanism could be related to an augmentation of the sympathetic autonomic activity after UCO (Ivankovich et al. 1974).

Surprising to us was the effect of ketamine on the magnitude of elevation in P_a_CO_2_ during the occlusion. The data in this study cannot fully explain this difference, although we do have some suggestions to possibly explain the mechanism. We first considered the possibility that, because ketamine pretreatment increased the arterial blood pressure during UCO, the umbilical‐placental perfusion might have been greater in that group. We had previously demonstrated that increases in fetal arterial pressure improve gas exchange across the placenta (Wood et al. 1989; Chen and Wood 1992). This interaction between blood pressure and gas exchange results from a lack of blood flow autoregulation by the placental vascular beds (Rudolph 1974; Paulick et al. 1991). Although the effect of increased blood pressure on placental gas exchange seemed to us to be an unlikely explanation (we were directly reducing umbilical‐placental perfusion with our occluder), we explored the relationship between arterial blood pressure and P_a_O_2_ or P_a_CO_2_. Neither of these correlations was statistically significant. The simplest explanation is that ketamine may have reduced the metabolic rate of the fetus. Reduced CO_2_ production rate would have theoretically reduced the magnitude of the increase in P_a_CO_2_ during UCO. We are not aware of any literature addressing the possible effects of ketamine on basal metabolic rate at any age. There are conflicting reports of ketamine effects on cerebral metabolic rate (Gaab et al. 1990; Akeson et al. 1993; Langsjo et al. 2003, 2005). Most of these studies reported an increase in cerebral oxygen consumption after ketamine treatment. Our own work involving transcriptomic modeling of fetal brain responses to maternal ventilatory hypoxia (producing fetal hypoxic hypoxia) indicated that the fetal hypothalamus reduced metabolic rate one hour after the onset of a 30‐min period of hypoxia (Wood et al. 2013). More recently, transcriptomics modeling indicated reduced metabolism in the fetal cerebral cortex – and reversal of that effect by ketamine – in the fetal cerebral cortex 24 h after the onset of 30‐min hypoxic hypoxia (Chang et al. 2016b). Interestingly, a similar modeling approach did not indicate any overall change in metabolism in the fetal kidney cortex 24 h after hypoxia (Chang et al. 2016a). While it would be difficult to explain a major change in CO_2_ production on the basis of altered metabolism in the fetal brain, we believe that the changes that we have observed in fetal brain may reflect a metabolic shift in other tissues during hypoxia (although not likely kidney), and we think it is possible that ketamine may reverse this effect in various tissues.

The fetal chemoreflex response to UCO and the subsequent effects on blood gases and hemodynamics is partially dependent on glutamate signaling mediated by NMDA receptors (Olney et al. 1986; Choi and Rothman 1990). Extracellular glutamate concentrations can increase after hypoxia secondary to neurotransmission and also because of reduced reuptake by neurons (Drejer et al. 1985; Choi and Rothman 1990). In other models of hypoxia, ketamine blockade of NMDA receptors reduced the ACTH responses to the stimuli (Makara and Stark 1975; Powers and Wood 2007; Chang and Wood 2015). Contrary to our original hypothesis, ketamine did not produce any effect on fetal ACTH concentrations compared to the control groups. It is possible that ketamine failed to block the ACTH response to UCO because of the hypercapnia, which is most likely sensed by central chemoreceptors and may not stimulate the HPA axis via glutamatergic neurotransmission.

**Figure 4:**
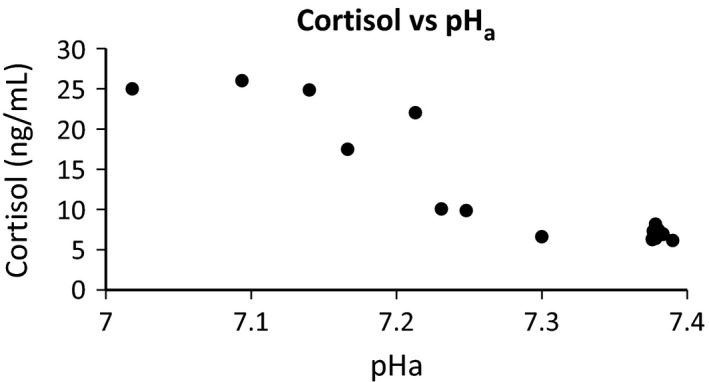
Fetal plasma cortisol concentration plotted as a function of arterial pH (pH_a_).

An unexpected aspect of the HPA response was that the fetal plasma cortisol responses to UCO were delayed in the ketamine‐treated group relative to the untreated group. This was not explained by the differences in fetal plasma ACTH concentration, so therefore may have resulted from other influences on adrenal cortical function: a decrease in cortisol clearance (Wood and Rudolph 1983; Dauprat et al. 1990) perhaps secondary to changes in hepatic blood flow, or possibly altered sympathetic nervous outflow to the adrenal cortex (McDonald and Nathanielsz 1998; Poore et al. 1998). In a previous study, we found that the fetal HPA axis is stimulated by acidification of fetal blood (Wood and Chen 1989). Later, we reported a statistically significant relationship between blood H^+^ concentrations and fetal cortisol concentrations, an interaction that was not apparently mediated by ACTH (Wood 2011). We speculated at the time of the later study that the fetal adrenal might be directly sensitive to acid, mediated by acid‐sensitive TWIK channels (Wood 2011). In the present experiments, fetal plasma cortisol and pH_a_ are highly correlated with each other (Fig. 4). If there were a direct effect of H^+^ on adrenal steroidogenesis in the fetus (as there is for aldosterone in the zona glomerulosa of the adult), the rise in cortisol concentrations would be most likely a response to the combined stimulus of rising plasma ACTH concentrations (rapidly rising to levels that saturate the adrenal response) and a modulation of that response by H^+^.

In summary, we report that ketamine modifies the cardiovascular response to UCO, but does not inhibit the ACTH response in late‐gestation fetal sheep. We conclude that the fetal ACTH response to UCO is not mediated solely by glutamatergic neurotransmission. We speculate, however, that ketamine decreases the metabolic rate of the fetal sheep.

## Perspectives

Umbilical cord occlusion is a common but potentially devastating event in human pregnancy. If severe, occlusion of the umbilical cord produces stroke‐like effects in the brain, an outcome results in tissue necrosis, apoptosis, and demyelination. Ketamine might spare brain function after fetal exposure to acute hypoxia observed during prolonged labor or insufficient uterine blood flow that could have short‐ and long‐term detrimental effects for the newborn.

## Conflict of Interests

None declared.
